# Safety and Efficacy of Fecal Microbiota, Live-jslm (REBYOTA^®^), for the Prevention of Recurrent *Clostridioides difficile* Infection in Participants With Inflammatory Bowel Disease in PUNCH CD3-OLS

**DOI:** 10.1093/ibd/izae291

**Published:** 2025-01-25

**Authors:** Jessica R Allegretti, Paul Feuerstadt, Whitfield L Knapple, Robert Orenstein, Philippe Pinton, Alexander Sheh, Sahil Khanna

**Affiliations:** Division of Gastroenterology, Hepatology and Endoscopy, Department of Medicine, Brigham & Women’s Hospital, Boston, MA, USA; Division of Digestive Disease, Yale University School of Medicine, New Haven, CT, USA; PACT-Gastroenterology Center, Hamden, CT, USA; Arkansas Gastroenterology, North Little Rock, AR, USA; Division of Infectious Diseases, Department of Internal Medicine, Mayo Clinic, Phoenix, AZ, USA; Global Research and Medical, Ferring Pharmaceuticals A/S, Kastrup, Denmark; Ferring Pharmaceuticals, Inc., Roseville, MN, USA; Department of Gastroenterology and Hepatology, Mayo Clinic, Rochester, MN, USA

**Keywords:** *C. difficile*, Crohn’s disease, microbiota restoration, RBX2660, ulcerative colitis

## Abstract

**Background:**

Fecal microbiota, live-jslm (RBL; REBYOTA^®^), is the first single-dose, broad consortia, microbiota-based live biotherapeutic approved by the US Food and Drug Administration to prevent recurrent *Clostridioides difficile* infection (rCDI) in adults following standard-of-care antimicrobials. Inflammatory bowel disease (IBD) is a common risk factor for rCDI, yet patients with IBD are often excluded from prospective trials. This subgroup analysis of PUNCH CD3-OLS (NCT03931941) evaluated the safety and efficacy of RBL in participants with rCDI and IBD.

**Methods:**

Participants with IBD (ulcerative colitis [UC], Crohn’s disease [CD], or unspecified) who had rCDI were included. Treatment-emergent adverse event (TEAE) data were collected for up to 6 months following RBL administration. Efficacy outcomes included treatment success at 8 weeks and sustained clinical response at 6 months.

**Results:**

Overall, 793 participants were enrolled, and 697 received RBL; 74 had IBD (UC: *n* = 45; CD: *n* = 25; unspecified IBD: *n* = 4). TEAEs within 8 weeks of administration were reported by 45.9% and 47.5% of participants with and without IBD, respectively; most were mild or moderate gastrointestinal symptoms. Serious TEAEs within 8 weeks of administration were reported by 1.4% and 4.2% of participants with and without IBD, respectively. The treatment success rate at 8 weeks was 78.9%, and the sustained clinical response rate at 6 months was 91.1% in participants with IBD, similar to rates in participants without IBD (73.2% and 91.0%, respectively).

**Conclusions:**

The results of this subgroup analysis of PUNCH CD3-OLS suggest RBL is safe and efficacious in patients with IBD.

**Key Messages**

**What is already known?**

- Individuals with inflammatory bowel disease (IBD) have an increased risk of developing recurrent *Clostridioides difficile* infection (rCDI) and experience worse clinical outcomes.

**What is new here?**

- This subgroup analysis of PUNCH CD3-OLS, which evaluated RBL safety and efficacy for prevention of rCDI, found RBL to be well-tolerated without marked safety signals in participants with IBD, with a treatment success rate of 78.9% at 8 weeks and sustained success through 6 months in 91.1% of participants.

**How can this study help patient care?**

- These findings support the use of RBL for prevention of rCDI in patients with rCDI and IBD.

## Introduction

*Clostridioides difficile* is the most common pathogen that causes healthcare-associated infection in the United States,^1^ affecting approximately half a million American patients annually.^2^ Compared with the general population, patients with inflammatory bowel disease (IBD; Crohn’s disease [CD] and ulcerative colitis [UC]) have a 4.8-fold increased risk of CDI^3^ and are more likely to experience recurrent *Clostridioides difficile* infection (rCDI).^4^ Approximately 2.4 million Americans have a diagnosis of IBD,^5^ which places a substantial burden on patients and healthcare systems.^6,7^ Factors that predispose patients with IBD to CDI and subsequent recurrences include disruption to the gut microbiome (eg, dysbiosis), exposure to immunosuppressive medications, frequent antibiotic use, and recurrent interaction with healthcare systems.^4,8,9^ The overlap in the clinical presentation of CDI and IBD makes the diagnosis and management of CDI in patients with IBD challenging, which is why screening for CDI is recommended at every disease flare in these patients.^8,10,11^ Individuals with CDI and IBD have worse clinical outcomes, including higher rates of colectomy, increased hospitalization, longer hospital stays, and increased mortality, compared with individuals without IBD.^4,12-15^ Therefore, appropriate management of both CDI and IBD in this vulnerable population is critical to minimize adverse outcomes.

Microbiota-based live biotherapeutic products have shown efficacy in preventing rCDI and help restore gut microbiome composition and diversity.^16,17^ Fecal microbiota, live-jslm (RBL; REBYOTA^®^, previously known as RBX2660), is the first single-dose, broad consortia, microbiota-based live biotherapeutic product approved by the US Food and Drug Administration (FDA) to prevent rCDI in adults following standard-of-care (SOC) antibiotic treatment.^18^ In PUNCH CD3 (NCT03244644), a prospective, randomized, double-blind, placebo-controlled Phase 3 trial, RBL demonstrated a greater treatment success rate compared with placebo (70.6% vs 57.5%).^16^ Moreover, RBL was well-tolerated, with mostly mild-to-moderate adverse events (AEs). However, patients commonly seen in real-world clinical settings, including those with IBD, have previously been excluded from prospective studies in the RBL clinical trial program, including the pivotal Phase 3 PUNCH CD3 trial.^16,19-21^

PUNCH CD3-OLS was an open-label study designed to assess the safety and efficacy of RBL for prevention of rCDI in a broad population that is reflective of patients seen in real-world clinical practice.^22^ In PUNCH CD3-OLS, RBL prevented CDI recurrence through 8 weeks in 73.8% of participants, with a sustained clinical response through 6 months in 91.0% of participants, consistent with the results of PUNCH CD3.^16^ This manuscript presents safety and efficacy data from an exploratory subgroup analysis of participants in PUNCH CD3-OLS with IBD.

## Methods

### Trial Design

PUNCH CD3-OLS (ClinicalTrials.gov, NCT03244644) was a Phase 3, open-label, single-arm, prospective study designed to evaluate the safety and tolerability of RBL in participants with rCDI. Each site obtained approval of the protocol from its institutional review board/research ethics board. The study was conducted in accordance with the ethical standards of the 1964 Declaration of Helsinki. Participants provided written informed consent prior to the initiation of any study procedures. Additional PUNCH CD3-OLS study details have been published previously.^22^

### Study Population

Participants included in this post hoc subgroup analysis had IBD, defined by a recorded medical history at the time of RBL administration of any of the following conditions: IBD, UC, or CD. If the treating clinician had recorded a medical history of “IBD” or both “UC” and “CD,” the participant was included in an “unspecified IBD” group. Concomitant use of IBD-related medication was at the discretion of the participant’s clinician and was not protocol specified.

Full inclusion/exclusion criteria have been published previously.^22^ Briefly, participants were aged ≥18 years old with documented rCDI as determined by the treating physician and confirmed use of SOC antibiotics or ≥2 episodes of severe CDI resulting in hospitalization. The method of rCDI diagnosis was not protocol specified; participants were diagnosed by the treating clinician, using methods available at the study site, or by the referring clinician. Participants had to be recently prescribed and/or taking SOC antibiotics for rCDI to control diarrhea at the time of enrollment. Participants’ diarrhea had to be controlled (<3 unformed/loose stools/day [Bristol Stool Scale type 6-7] for the 2 consecutive days prior to the 24- to 72-hour antibiotic washout period). Exclusion criteria included history of continued CDI diarrhea despite antibiotic treatment, use of non-CDI antibiotics, and prior participation in a clinical study evaluating RBL.

### Study Treatment

Participants received a single 150-mL dose of RBL administered rectally within 24-72 hours of completing SOC antibiotics. Each dose contained between 1 × 10^8^ and 5 × 10^10^ colony-forming units (CFUs)/mL of fecal microbes. No bowel preparation or colonoscopy was required prior to or for RBL administration. During the 24- to 72-hour period before RBL administration, SOC antibiotics were not permitted to allow for the wash out of antibiotic treatments that have a potential impact on RBL-related outcomes. Participants were eligible to receive a second course of RBL within 21 calendar days if they met the protocol-specified criteria for treatment failure, as described below. The use of antibiotics prior to a second course of RBL administration was at the discretion of the investigator. If antibiotics were given to control symptoms, a 24- to 72-hour washout period was required prior to administration of the second RBL course.

### Study Outcomes

The number of participants with RBL- or administration-related treatment-emergent AEs (TEAEs) was assessed. TEAEs were defined as any AE occurring on the same day or after RBL administration and were coded using the Medical Dictionary for Regulatory Activities (MedDRA), version 20.0. The severity of TEAEs, relatedness of TEAEs, and the number of select CDI-related complications through 8 weeks after RBL administration were assessed. For a preexisting condition to be recorded as an AE, there had to be a worsening in its frequency, intensity, or character on the day of or after RBL administration. Safety data were censored at CDI occurrence.

Efficacy outcomes included treatment success and sustained clinical response. Treatment success was defined as the absence of CDI diarrhea (ie, 3 or more unformed stools in 24 or fewer consecutive hours for at least 2 consecutive days) through 8 weeks after RBL administration. Sustained clinical response was defined as treatment success of the presenting rCDI and no new episodes of CDI through 6 months after RBL administration. Treatment failure (CDI recurrence) was defined as the presence of CDI diarrhea within 8 weeks of RBL administration. Recurrence was confirmed by a positive stool test in a central laboratory (C. Diff Quik Chek Complete^®^ Test; enzyme immunoassay test for glutamate dehydrogenase [GDH] and toxins A and B). Polymerase chain reaction–based testing was carried out following discordant results (eg, GDH-positive and toxin-negative) to confirm treatment failure.

Concomitant medications were documented and categorized using the Anatomical Therapeutic Chemical (ATC) classification system. For the analysis of concomitant medication usage by participants with UC or CD, a subset of concomitant medications relevant to the treatment of IBD was selected based on the fourth level of the ATC codes. Concomitant medication classes included in the analysis are as follows: aminosalicylic acid and similar agents (mesalazine and balsalazide); corticosteroids acting locally (budesonide); glucocorticoids (prednisone, methylprednisolone, and hydrocortisone); interleukin inhibitors (ustekinumab); other immunosuppressants (methotrexate); purine analogs (azathioprine and mercaptopurine); selective immunosuppressants (vedolizumab and tofacitinib); and tumor necrosis factor-alpha (TNF-α) inhibitors (infliximab and adalimumab). Concomitant medication logs were evaluated to determine if any medication (prescription or nonprescription) in the defined classes was taken or used from the time of RBL administration (baseline) through the 6-month follow-up. Participants with ongoing treatment with at least 1 medication within the defined classes at the specified time periods were considered as treated with concomitant medications for IBD. For analysis of individual drug classes, a participant receiving multiple drugs within the same class at a given time point (eg, treatment with oral and rectal mesalazine at 8 weeks) was counted as a single entry. Participants treated with medications in multiple classes were counted within each class.

### Statistical Analysis

Descriptive statistics were reported for all outcomes. The analysis of safety was performed on the safety population, defined as all participants who had been exposed to RBL. The intent-to-treat (ITT) population was defined as all participants enrolled in the study. Efficacy analyses were performed on the modified ITT (mITT) population, defined as all participants who received RBL but excluding those in whom administration was attempted but not completed and those who discontinued the study prior to evaluation of treatment success or failure.

## Results

### Participants

**Figure 1.**
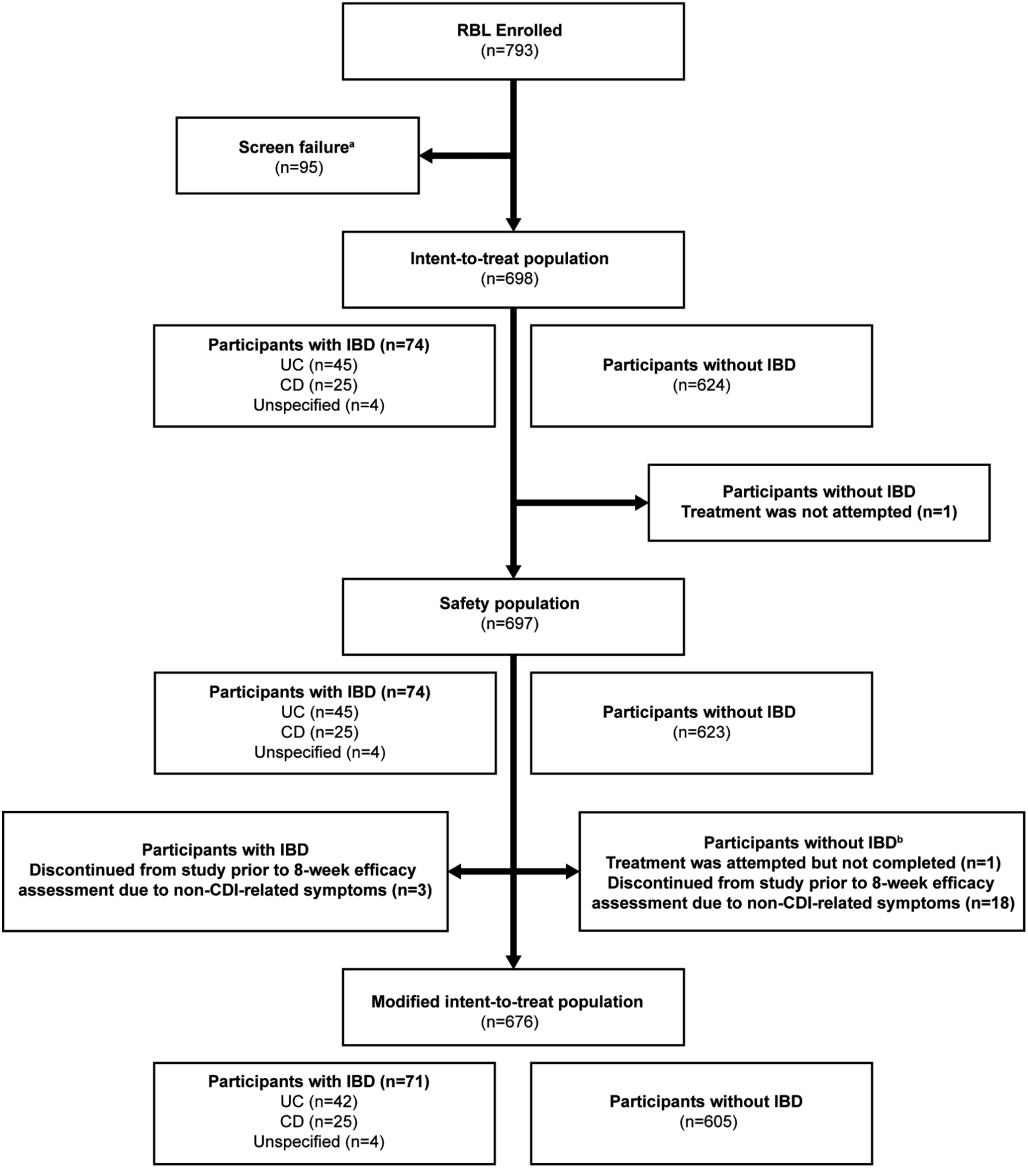
CONSORT diagram showing enrollment and subgroups. ^a^Participants who had signed informed consent forms but did not meet all eligibility criteria were considered screen failures. ^b^Participants could have >1 reason for exclusion from the modified intent-to-treat population. Abbreviations: CD, Crohn’s disease; CDI, *Clostridioides difficile* infection; IBD, inflammatory bowel disease; RBL, fecal microbiota, live-jslm; UC, ulcerative colitis.

Overall, 793 participants were enrolled in PUNCH CD3-OLS and 697 participants received RBL. Of these, 74 participants had documented IBD, including 45 with UC and 25 with CD; 4 participants had unspecified IBD. Three participants with UC were excluded from the mITT population due to study withdrawal (*n* = 2) and loss to follow-up (*n* = 1) prior to the 8-week assessment (Figure 1).

Participants with and without IBD had a median (interquartile range) age of 47.0 (38.0, 66.0) and 65.0 (51.0, 74.0) years, respectively (Table 1). The majority (74.3%) of participants with IBD were aged <65 years. There were fewer female participants with IBD compared with those without IBD (with IBD: 55.4% female; without IBD: 71.6% female). Other baseline characteristics were well-balanced between groups. Most participants with and without IBD had previously received vancomycin for the enrolling rCDI episode (87.8% and 85.1%, respectively). Overall, 92.0% (23/25) of participants with CD and 75.6% (34/45) of participants with UC were taking concomitant IBD-related medication at any time during the study (Table 2). The number of participants taking concomitant IBD-related medication was comparable over time (baseline, 8 weeks, and 6 months after RBL administration).

**Table 1.** Participant demographics and baseline characteristics (safety population).

	With IBD *N* = 74	Without IBD *N* = 623
Age		
Median (IQR)	47.0 (38.0, 66.0)	65.0 (51.0, 74.0)
<65 y, *n* (%)	55 (74.3)	304 (48.8)
≥65 y, *n* (%)	19 (25.7)	319 (51.2)
Sex, *n* (%)		
Female	41 (55.4)	446 (71.6)
Race, *n* (%)		
White	67 (90.5)	587 (94.2)
Non-White	7 (9.5)	36 (5.8)
Ethnicity, *n* (%)		
Hispanic or Latino	4 (5.4)	11 (1.8)
Not Hispanic or Latino	68 (91.9)	602 (96.6)
Not reported or unknown	2 (2.7)	10 (1.6)
Number of previous CDI episodes, *n* (%)^a^		
2	23 (31.1)	163 (26.2)
3	27 (36.5)	241 (38.7)
≥4	23 (31.1)	216 (34.7)
Most recent CDI antibiotics, *n* (%)		
Vancomycin	65 (87.8)	530 (85.1)
Fidaxomicin	9 (12.2)	89 (14.3)
Rifaximin	0	7 (1.1)
Charlson Comorbidity Index, mean (SD)	1.9 (2.34)	3.3 (2.74)

Abbreviations: CDI, *Clostridioides difficile* infection; IBD, inflammatory bowel disease; IQR, interquartile range.

^a^Number of CDI episodes recorded was incomplete for 4 participants.

**Table 2.** Summary of concomitant IBD-related medications at the time of RBL administration and 8 weeks and 6 months after administration (safety population).

	CD *N* = 25			UC *N* = 45		
Participants treated with concomitant medication, *n* (%)^a^	23 (92.0)			34 (75.6)		
Drug classes, *n* (%)^a^	Baseline	8 wk	6 mo	Baseline	8 wk	6 mo
ASA and similar agents^b^	3 (12.0)	3 (12.0)	5 (20.0)	19 (42.2)	19 (42.2)	21 (46.7)
Corticosteroids acting locally^c^	4 (16.0)	3 (12.0)	3 (12.0)	2 (4.4)	3 (6.7)	4 (8.9)
Glucocorticoids^d^	4 (16.0)	7 (28.0)	7 (28.0)	6 (13.3)	9 (20.0)	7 (15.6)
Immunosuppressants						
Tofacitinib	0	0	0	1 (2.2)	1 (2.2)	1 (2.2)
Vedolizumab	4 (16.0)	2 (8.0)	2 (8.0)	8 (17.8)	8 (17.8)	10 (22.2)
Interleukin inhibitors^e^	3 (12.0)	3 (12.0)	4 (16.0)	3 (6.7)	3 (6.7)	4 (8.9)
Other immunosuppressants^f^	2 (8.0)	1 (4.0)	1 (4.0)	1 (2.2)	2 (4.4)	1 (2.2)
Purine analogs^g^	4 (16.0)	4 (16.0)	4 (16.0)	0	0	0
TNF-α inhibitors^h^	4 (16.0)	5 (20.0)	5 (20.0)	9 (20.0)	9 (20.0)	8 (17.8)

Abbreviations: ASA, aminosalicylic acid; CD, Crohn’s disease; IBD, inflammatory bowel disease; RBL, fecal microbiota, live-jslm; TNF, tumor necrosis factor; UC, ulcerative colitis.

^a^Participants could be on >1 IBD-related medication.

^b^Mesalazine and balsalazide.

^c^Budesonide.

^d^Prednisone, methylprednisolone, and hydrocortisone. Three participants with UC and 4 participants with CD had recorded doses of prednisone or methylprednisolone over 20 mg.

^e^Ustekinumab.

^f^Methotrexate.

^g^Azathioprine and mercaptopurine.

^h^Infliximab and adalimumab.

### Safety

Within 8 weeks of RBL administration, 45.9% (34/74) and 47.5% (296/623) of participants with and without IBD reported TEAEs, respectively (Table 3). Within the IBD subgroup, 52.0% (13/25) of participants with CD and 37.8% (17/45) of participants with UC reported TEAEs, with most reporting TEAEs of mild (IBD: 17.6%; CD: 20.0%; UC: 11.1%; no IBD: 20.7%) and moderate (IBD: 20.3%; CD: 28.0%; UC: 15.6%; no IBD: 19.7%) severity. Most TEAEs in participants with and without IBD were related to preexisting conditions (23.0% and 23.1%, respectively). Between 8 weeks and 6 months of follow-up, 24.3% (18/74) and 22.8% (142/623) of participants with and without IBD reported TEAEs, respectively. Within the IBD group, 28.0% (7/25) of participants with CD and 22.2% (10/45) of participants with UC reported TEAEs.

**Table 3.** Summary of TEAEs within the 8-week and between the 8-week and 6-month follow-up periods of RBL administration (safety population).

	Within 8 wk (*N* = 697)				8 wk to 6 mo (*N* = 697)			
	With IBD *N* = 74	CD^a^ *N* = 25	UC^a^ *N* = 45	Without IBD *N* = 623	With IBD *N* = 74	CD^a^ *N* = 25	UC^a^ *N* = 45	Without IBD *N* = 623
	Participants (% of participants) [events]							
Any TEAE	34 (45.9) [79]	13 (52.0) [29]	17 (37.8) [44]	296 (47.5) [715]	18 (24.3) [34]	7 (28.0) [11]	10 (22.2) [22]	142 (22.8) [297]
TEAEs by maximum severity								
Mild	13 (17.6) [45]	5 (20.0) [16]	5 (11.1) [24]	129 (20.7) [411]	3 (4.1) [8]	1 (4.0) [4]	1 (2.2) [3]	48 (7.7) [133]
Moderate	15 (20.3) [27]	7 (28.0) [12]	7 (15.6) [14]	123 (19.7) [234]	13 (17.6) [19]	6 (24.0) [7]	7 (15.6) [12]	66 (10.6) [124]
Severe	6 (8.1) [7]	1 (4.0) [1]	5 (11.1) [6]	40 (6.4) [66]	2 (2.7) [7]	0	2 (4.4) [7]	24 (3.9) [34]
Potentially life-threatening	0	0	0	4 (0.6) [4]	0	0	0	4 (0.6) [6]
TEAEs by relatedness^b^								
Related to RBL	9 (12.2) [22]	1 (4.0) [1]	6 (13.3) [19]	97 (15.6) [176]	0	0	0	4 (0.6) [4]
Related to administration procedure	7 (9.5) [12]	1 (4.0) [1]	4 (8.9) [9]	53 (8.5) [84]	0	0	0	1 (0.2) [2]
Related to CDI	7 (9.5) [11]	2 (8.0) [2]	4 (8.9) [8]	59 (9.5) [90]	1 (1.4) [1]	0	1 (2.2) [1]	11 (1.8) [16]
Related to a preexisting condition	17 (23.0) [27]	7 (28.0) [13]	8 (17.8) [10]	144 (23.1) [266]	9 (12.2) [13]	3 (12.0) [4]	5 (11.1) [8]	59 (9.5) [96]
Any serious TEAE	1 (1.4) [1]	0	1 (2.2) [1]	26 (4.2) [34]	3 (4.1) [4]	1 (4.0) [2]	2 (4.4) [2]	22 (3.5) [33]
Serious TEAEs by relatedness^b^								
Related to RBL	1 (1.4)^c^ [1]	0	1 (2.2)^c^ [1]	0	0	0	0	0
Related to administration procedure	0	0	0	0	0	0	0	0
Related to CDI	0	0	0	1 (0.2) [1]	0	0	0	1 (0.2) [1]
Related to a preexisting condition	1 (1.4)^c^ [1]	0	1 (2.2)^c^ [1]	18 (2.9) [22]	2 (2.7) [3]	1 (4.0) [2]	1 (2.2) [1]	16 (2.6) [22]
TEAEs leading to withdrawal from study	0	0	0	4 (0.6) [4]	0	0	0	1 (0.2) [1]
TEAEs leading to death	0	0	0	3 (0.5) [3]	0	0	0	1 (0.2) [1]

Abbreviations: CD, Crohn’s disease; CDI, *Clostridioides difficile* infection; IBD, inflammatory bowel disease; RBL, fecal microbiota, live-jslm; TEAE, treatment-emergent adverse event; UC, ulcerative colitis.

^a^CD and UC are subtypes of IBD. Four participants with unspecified IBD were excluded from the safety analysis.

^b^Relatedness categories are not mutually exclusive.

^c^A flare of preexisting UC (assessed as definitely related to a preexisting condition and possibly related to RBL).

The most common TEAEs throughout the study period were gastrointestinal, reported in 35.1% of participants with IBD (CD: 36.0%; UC: 31.1%) and 32.7% of participants without IBD (Table 4). The most common gastrointestinal symptoms were diarrhea (CD: 24.0%; UC: 13.3%; without IBD: 13.5%), abdominal pain (CD: 12.0%; UC: 15.6%; without IBD: 10.4%), and nausea (CD: 12.0%; UC: 13.3%; without IBD: 5.6%). In the IBD subgroup, 3 participants experienced a flare-up or worsening of their preexisting UC within 8 weeks of RBL administration.

**Table 4.** TEAEs by system organ class and preferred term with an incidence of ≥5% following RBL administration^a^ (overall study period; safety population).

	With IBD *N* = 74	CD^b^ *N* = 25	UC^b^ *N* = 45	Without IBD *N* = 623
	Participants (% of participants) [events]			
Gastrointestinal disorders	26 (35.1) [55]	9 (36.0) [17]	14 (31.1) [34]	204 (32.7) [431]
Diarrhea	13 (17.6) [14]	6 (24.0) [6]	6 (13.3) [7]	84 (13.5) [99]
Abdominal pain	11 (14.9) [14]	3 (12.0) [4]	7 (15.6) [9]	65 (10.4) [72]
Nausea	9 (12.2) [9]	3 (12.0) [3]	6 (13.3) [6]	35 (5.6) [40]
Abdominal distension	3 (4.1) [3]	NA^c^	NA^c^	36 (5.8) [45]
UC flare	3 (4.1) [3]^d^	0	3 (6.7) [3]^d^	0
Infections	14 (18.9) [17]	7 (28.0) [9]	7 (15.6) [8]	114 (18.3) [142]
Urinary tract infection	4 (5.4) [5]	1 (4.0) [2]	3 (6.7) [3]	19 (3.0) [21]

Coding was based on MedDRA version 20.0. Abbreviations: CD, Crohn’s disease; IBD, inflammatory bowel disease; MedDRA, Medical Dictionary for Regulatory Activities; NA, not assessed; RBL, fecal microbiota, live-jslm; TEAE, treatment-emergent adverse event; UC, ulcerative colitis.

^a^TEAEs are reported where participants in any subgroup had an incidence ≥5%, listed in order of incidence in the IBD group.

^b^CD and UC are subtypes of IBD. Four participants with unspecified IBD were excluded from the safety analysis by subtype.

^c^TEAEs occurred in the subgroup at an incidence of <5%.

^d^All 3 participants experienced a flare-up or worsening of their preexisting UC within 8 wk of RBL administration; 1 event was considered a serious TEAE (assessed as definitely related to a preexisting condition and possibly related to RBL).

Serious TEAEs were reported in 1.4% (1/74) of participants with IBD and 4.2% (26/623) of participants without IBD within 8 weeks of RBL administration (Table 3). One participant with UC experienced a flare, classified as a serious TEAE, which was assessed as definitely related to a preexisting condition and possibly related to RBL. Between 8 weeks and 6 months of follow-up, serious TEAEs were reported in 4.1% (3/74) of participants with IBD (CD: 4.0%; UC: 4.4%) and 3.5% (22/623) of participants without IBD. In participants with and without IBD, most serious TEAEs were related to a preexisting condition: within 8 weeks, 1.4% and 2.9%, respectively; between 8 weeks and 6 months, 2.7% and 2.6%, respectively. No participants with IBD reported complications related to CDI recurrence following RBL administration (eg, emergency colectomy). One participant with IBD received surgery during the study (laparoscopic hernia). No participants with IBD withdrew from the study due to TEAEs. There were no deaths in participants with IBD. Four deaths, unrelated to RBL, occurred in participants without IBD (cardiac arrest, cardiac failure, spina bifida, and pulmonary sepsis).

### Efficacy

**Figure 2.**
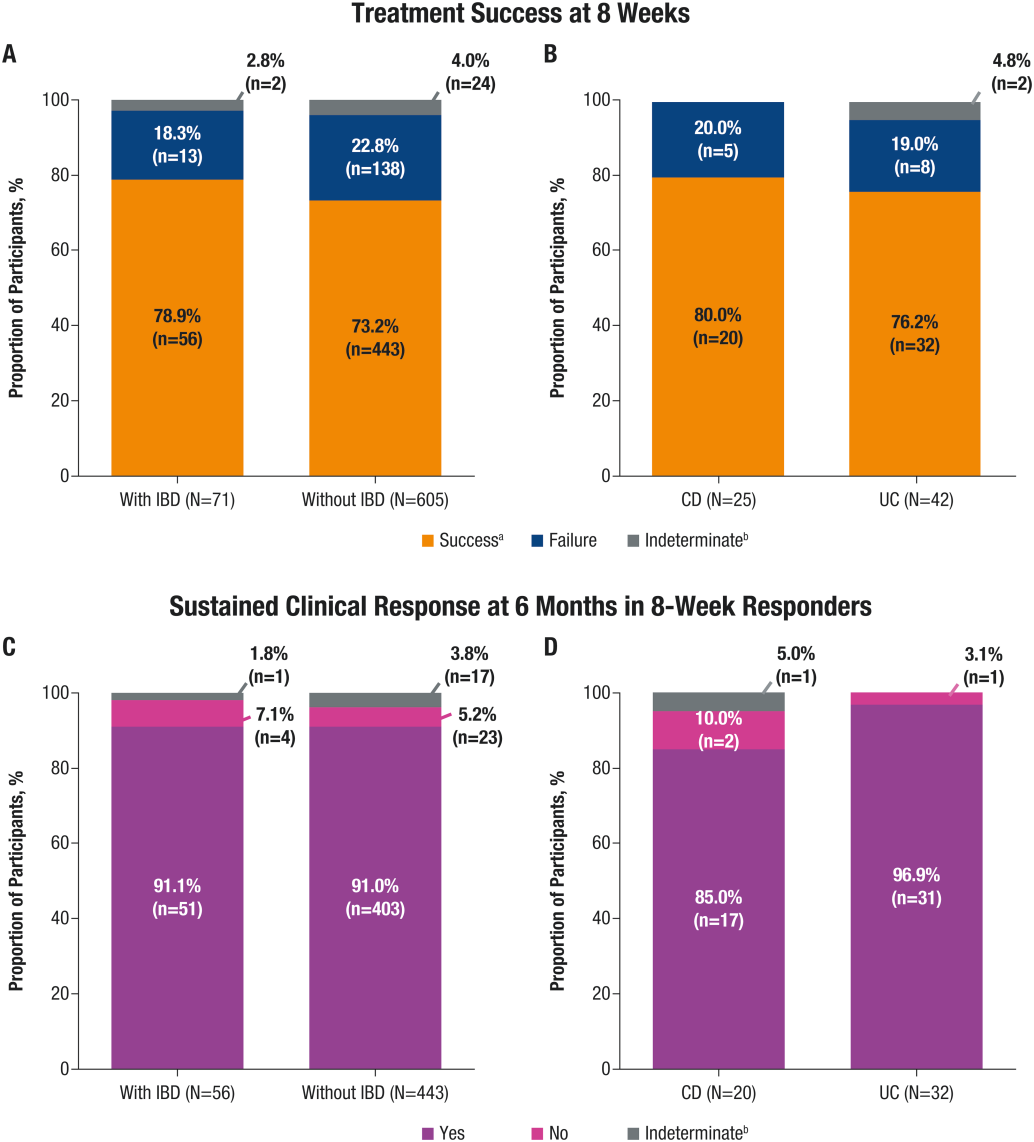
Efficacy outcomes within 8 wk and between 8 wk and 6 mo of RBL administration (mITT population). Treatment success within 8 wk of RBL administration in (A) participants with and without IBD and (B) participants with CD and UC (IBD subgroups). Sustained clinical response at 6 mo in 8-wk responders in (C) participants with and without IBD and (D) participants with CD and UC (IBD subgroups). ^a^Treatment success is defined as the absence of CDI-related diarrhea through 8 wk after RBL administration. ^b^Efficacy outcome was indeterminate if CDI test was inconclusive or missed at time of visit. Abbreviations: CD, Crohn’s disease; CDI, *Clostridioides difficile* infection; IBD, inflammatory bowel disease; RBL, fecal microbiota, live-jslm; mITT, modified intent-to-treat; UC, ulcerative colitis.

In the mITT population, treatment success was achieved in 78.9% (56/71) of participants with IBD and 73.2% (443/605) without IBD (Figure 2A). In participants with IBD, treatment success was achieved in 80.0% (20/25) and 76.2% (32/42) of those with CD and UC, respectively (Figure 2B). Of the participants who achieved treatment success, 91.1% (51/56) and 91.0% (403/443) of those with and without IBD, respectively, had a sustained clinical response at 6 months (Figure 2C). Sustained clinical response at 6 months was achieved by 85.0% (17/20) and 96.9% (31/32) of participants with CD and UC, respectively (Figure 2D). In time to failure analysis, the mean (SD) time to failure of RBL was 15.8 days (±16.9 days) in participants with IBD and 17.0 days (±15.8 days) in participants without IBD.

### Second Course of RBL

Of the 13 (CD: *n* = 5; UC: *n* = 8) participants with IBD and 138 participants without IBD who met the criteria for treatment failure, 9 with IBD and 112 without IBD opted to receive a second course of RBL within 21 days of treatment failure. Overall, 12.7% (9/71) of participants with IBD and 18.5% (112/605) of participants without IBD received 2 courses of RBL.

Within 8 weeks of second course administration, 22.2% (2/9) and 40.2% (45/112) of participants with and without IBD reported TEAEs, respectively (Supplementary Table 1). Most TEAEs following a second course were mild to moderate in severity. No serious TEAEs were assessed as related to RBL or its administration.

Following a second course of RBL, 22.2% (2/9) of participants with IBD and 58.0% (65/112) without IBD had treatment success (Supplementary Table 2). Of the participants who had treatment success following a second course of RBL, 100% (2/2) and 87.7% (57/65) of participants with and without IBD, respectively, had a sustained clinical response at 6 months. Overall, 81.7% (58/71) and 84.0% (508/605) of participants with and without IBD, respectively, achieved treatment success in the IBD and no IBD groups, regardless of the number of RBL administrations.

## Discussion

PUNCH CD3-OLS, the largest open-label study of a broad consortia microbiota-based live biotherapeutic to date, evaluated the safety and efficacy of RBL in a real-world population that included participants at high risk of rCDI who are usually excluded from randomized clinical trials.^22^ Here, findings are reported for 74 participants with IBD who were administered RBL following rCDI. This is the first study to report on the safety and efficacy of an FDA-approved microbiota-based live biotherapeutic in patients with concurrent rCDI and IBD.

In this study, RBL was efficacious and safe for the prevention of rCDI in participants with IBD. The treatment success rate in participants with rCDI and concurrent IBD at 8 weeks was 79%, with sustained clinical response through 6 months observed in 91% of participants. These results were comparable to the 73% treatment success rate and 91% sustained clinical response rate observed in participants without IBD. Treatment success in participants with IBD was numerically higher than the 71% treatment success rate reported in the pivotal Phase 3 clinical trial, PUNCH CD3, which excluded participants with IBD and other comorbid conditions.^16^ This finding aligns with a recent meta-analysis which found that efficacy outcomes following microbiota restoration are higher in open-label trials compared with randomized controlled trials, likely due to factors such as strict inclusion/exclusion criteria and end point definitions.^23^ The sustained clinical response rate through 6 months in participants with IBD who achieved treatment success in PUNCH CD3-OLS was similar to the PUNCH CD3 population (91% vs 92%, respectively).^16^

Previous studies using investigational fecal microbiota transplantation (FMT) can provide historical context to the PUNCH CD3-OLS results. In a systematic review of FMT use in patients with both CDI and IBD, clinical CDI resolution rates after 1 dose of FMT ranged from 53% to 83%, with a calculated^24^ weighted pooled cure rate of 78%.^25^ While a previous retrospective study found that IBD lowered the efficacy of FMT to prevent rCDI,^26^ a meta-analysis of cohort studies found no significant difference in CDI prevention rates between patients with and without IBD.^27^ In this subgroup analysis, presence of IBD did not reduce the efficacy of RBL in preventing rCDI.

In this study, most participants with IBD received 1 course of RBL, with 13% of participants receiving 2 courses of RBL after initial treatment failure. Out of the 9 participants with IBD who received a second course of RBL, 2 had treatment success. However, 4 participants had indeterminate results. Both participants who experienced treatment success after the second course of RBL maintained a sustained clinical response at the 6-month follow-up. Overall, 82% of participants with IBD had treatment success, regardless of the number of RBL administrations. Ultimately, interpretation of these data is limited due to the small number of participants receiving a second dose of RBL. While a subset of patients may benefit from multiple doses of RBL, future research is required to elucidate the factors contributing to this effect.

RBL was found to be well-tolerated in participants with IBD, without marked safety signals. The proportion of participants who reported TEAEs within 8 weeks and between 8 weeks and 6 months of RBL administration was comparable between participants with and without IBD. Most TEAEs in both groups were mild to moderate in severity, with diarrhea and abdominal pain being the most common. These results are consistent with safety data from the overall PUNCH CD3-OLS and previous studies in the RBL clinical trial program, which had up to 2 years of follow-up.^22,28^ TEAEs were reported by a similar proportion of participants in the CD and UC subgroups.

Although colectomy following FMT has been reported in previous studies,^25^ no participants in PUNCH CD3-OLS underwent an emergency colectomy. One serious TEAE (UC flare) was reported as definitely related to a preexisting condition in a participant with severe UC and as possibly related to the use of RBL. Overall, flare or worsening of existing UC was reported in 3 patients with UC (3/45, 6.7%). IBD flares have been reported previously in patients with IBD and CDI following administration of FMT for the prevention of CDI.^25,29^ Meta-analyses have reported pooled rates of IBD flares and/or worsening following FMT for CDI in patients with IBD as 22.7% and 26.8%.^25,29^ However, in a prospective trial assessing the effect of FMT in patients with IBD and CDI, 4% (*n* = 1) of the UC subgroup had a de novo IBD flare.^30^ Ultimately, the rate of IBD worsening following FMT is influenced by reporting bias in small retrospective studies. Furthermore, definitions of flare or IBD worsening differ between studies (eg, hospitalization, escalation of therapy, and/or worsening symptoms) or are lacking altogether within those trials.^25,29^

Interpretation of these study findings requires several considerations. This open-label study was designed to evaluate the safety of RBL in a real-world population with rCDI. Therefore, participants without IBD may have had other relevant comorbidities that are not detailed in this analysis. As the trial evaluated a patient population with rCDI and a broad range of comorbid medical conditions, no direct assessment of IBD activity was required. Therefore, several data points or outcomes of clinical interest in an IBD population were not captured. Similarly, AEs were used as proxies for changes in IBD. Future studies will help validate the findings of this exploratory subgroup analysis.

This analysis represents the first report of the safety and efficacy of an FDA-approved microbiota-based live therapeutic in patients with rCDI and concurrent IBD. The broad patient population included in PUNCH CD3-OLS allowed for analysis of a large subgroup of participants with IBD and enabled a robust analysis.

In conclusion, the results of this post hoc subgroup analysis suggest RBL can be used safely and is efficacious in patients with rCDI and IBD. Outcomes for participants with IBD were similar to those of the overall population and aligned with findings from a meta-analysis of conventional FMT in similar populations,^25^ which are recommended in current clinical guidelines.^31,32^ Additionally, rectally administered RBL avoids the costs and risks associated with another colonoscopy in patients with IBD. Given that patients with IBD are at high risk of CDI and have worse clinical outcomes than patients without IBD, the results from PUNCH CD3-OLS demonstrate that RBL has promising potential to prevent rCDI in this vulnerable population, without appreciable risk.

## Supplementary Data

Supplementary data are available at *Inflammatory Bowel Diseases* online.

izae291_suppl_Supplementary_Tables_S1-S2

## Data Availability

Ferring Pharmaceuticals, Inc., will provide access to individual de-identified participant data upon request, via a secure portal, to researchers whose proposals meet the research criteria and other conditions. To gain access, data requestors must enter into a data access agreement with Ferring Pharmaceuticals Inc.

## References

[1] Magill SS , O’Leary E, Janelle SJ, et al; Emerging Infections Program Hospital Prevalence Survey Team. Changes in prevalence of health care-associated infections in U.S. hospitals. N Engl J Med. 2018;379(18):1732-1744. doi: https://doi.org/10.1056/NEJMoa1801550 30380384 PMC7978499

[2] Feuerstadt P , Theriault N, Tillotson G. The burden of CDI in the United States: a multifactorial challenge. BMC Infect Dis. 2023;23(1):132. doi: https://doi.org/10.1186/s12879-023-08096-0 36882700 PMC9990004

[3] Singh H , Nugent Z, Yu BN, Lix LM, Targownik LE, Bernstein CN. Higher incidence of *Clostridium difficile* infection among individuals with inflammatory bowel disease. Gastroenterology. 2017;153(2):430-438.e2. doi: https://doi.org/10.1053/j.gastro.2017.04.044 28479377

[4] Razik R , Rumman A, Bahreini Z, McGeer A, Nguyen GC. Recurrence of *Clostridium difficile* infection in patients with inflammatory bowel disease: the RECIDIVISM study. Am J Gastroenterol. 2016;111(8):1141-1146. doi: https://doi.org/10.1038/ajg.2016.187 27215924

[5] Lewis JD , Parlett LE, Jonsson Funk ML, et al Incidence, prevalence, and racial and ethnic distribution of inflammatory bowel disease in the United States. Gastroenterology. 2023;165(5):1197-1205.e2. doi: https://doi.org/10.1053/j.gastro.2023.07.003 37481117 PMC10592313

[6] Charabaty A , Schneider B, Zambrano JA, Keefer L. Living with inflammatory bowel disease: online surveys evaluating patient perspectives on treatment satisfaction and health-related quality of life. Crohns Colitis 360. 2022;4(3):otac035. doi: https://doi.org/10.1093/crocol/otac035 36777425 PMC9802169

[7] Singh S , Qian AS, Nguyen NH, et al Trends in U.S. health care spending on inflammatory bowel diseases, 1996-2016. Inflamm Bowel Dis. 2022;28(3):364-372. doi: https://doi.org/10.1093/ibd/izab074 33988697 PMC8889287

[8] Dalal RS , Allegretti JR. Diagnosis and management of *Clostridioides difficile* infection in patients with inflammatory bowel disease. Curr Opin Gastroenterol. 2021;37(4):336-343. doi: https://doi.org/10.1097/MOG.0000000000000739 33654015 PMC8169557

[9] Schneeweiss S , Korzenik J, Solomon DH, Canning C, Lee J, Bressler B. Infliximab and other immunomodulating drugs in patients with inflammatory bowel disease and the risk of serious bacterial infections. Aliment Pharmacol Ther. 2009;30(3):253-264. doi: https://doi.org/10.1111/j.1365-2036.2009.04037.x 19438424

[10] Khanna S , Shin A, Kelly CP. Management of *Clostridium difficile* infection in inflammatory bowel disease: expert review from the Clinical Practice Updates Committee of the AGA Institute. Clin Gastroenterol Hepatol. 2017;15(2):166-174. doi: https://doi.org/10.1016/j.cgh.2016.10.024 28093134

[11] Kucharzik T , Ellul P, Greuter T, et al ECCO guidelines on the prevention, diagnosis, and management of infections in inflammatory bowel disease. J Crohns Colitis. 2021;15(6):879-913. doi: https://doi.org/10.1093/ecco-jcc/jjab052 33730753

[12] Berg AM , Kelly CP, Farraye FA. *Clostridium difficile* infection in the inflammatory bowel disease patient. Inflamm Bowel Dis. 2013;19(1):194-204. doi: https://doi.org/10.1002/ibd.22964 22508484

[13] Chen Y , Furuya-Kanamori L, Doi SA, Ananthakrishnan AN, Kirk M. *Clostridium difficile* infection and risk of colectomy in patients with inflammatory bowel disease: a bias-adjusted meta-analysis. Inflamm Bowel Dis. 2017;23(2):200-207. doi: https://doi.org/10.1097/MIB.0000000000000998 28079620

[14] Law CC , Tariq R, Khanna S, Murthy S, McCurdy JD. Systematic review with meta-analysis: the impact of *Clostridium difficile* infection on the short- and long-term risks of colectomy in inflammatory bowel disease. Aliment Pharmacol Ther. 2017;45(8):1011-1020. doi: https://doi.org/10.1111/apt.13972 28206678

[15] Tariq R , Law CCY, Khanna S, Murthy S, McCurdy JD. The impact of *Clostridium difficile* infection on mortality in patients with inflammatory bowel disease: a systematic review and meta-analysis. J Clin Gastroenterol. 2019;53(2):127-133. doi: https://doi.org/10.1097/MCG.0000000000000968 29206751

[16] Khanna S , Assi M, Lee C, et al Efficacy and safety of RBX2660 in PUNCH CD3, a phase III, randomized, double-blind, placebo-controlled trial with a Bayesian primary analysis for the prevention of recurrent *Clostridioides difficile* infection. Drugs. 2022;82(15):1527-1538. doi: https://doi.org/10.1007/s40265-022-01797-x 36287379 PMC9607700

[17] Blount KF , Shannon WD, Deych E, Jones C. Restoration of bacterial microbiome composition and diversity among treatment responders in a phase 2 trial of RBX2660: an investigational microbiome restoration therapeutic. Open Forum Infect Dis. 2019;6(4):ofz095. doi: https://doi.org/10.1093/ofid/ofz095 31024971 PMC6475591

[18] REBYOTA® [Prescribing Information]. Parsippany, NJ: Ferring Pharmaceuticals Inc.; 2022.

[19] Dubberke ER , Orenstein R, Khanna S, Guthmueller B, Lee C. Final results from a phase 2b randomized, placebo-controlled clinical trial of RBX2660: a microbiota-based drug for the prevention of recurrent *Clostridioides difficile* infection. Infect Dis Ther. 2023;12(2):703-709. doi: https://doi.org/10.1007/s40121-022-00744-3 36544075 PMC9925615

[20] Langdon A , Schwartz DJ, Bulow C, et al; CDC Prevention Epicenter Program. Microbiota restoration reduces antibiotic-resistant bacteria gut colonization in patients with recurrent *Clostridioides difficile* infection from the open-label PUNCH CD study. Genome Med. 2021;13(1):28. doi: https://doi.org/10.1186/s13073-021-00843-9 33593430 PMC7888090

[21] Orenstein R , Dubberke ER, Khanna S, et al Durable reduction of *Clostridioides difficile* infection recurrence and microbiome restoration after treatment with RBX2660: results from an open-label phase 2 clinical trial. BMC Infect Dis. 2022;22(1):245. doi: https://doi.org/10.1186/s12879-022-07256-y 35279084 PMC8917640

[22] Feuerstadt P , Chopra T, Knapple W, et al PUNCH CD3-OLS: a phase 3 prospective observational cohort study to evaluate the safety and efficacy of fecal microbiota, live-jslm (REBYOTA) in adults with recurrent *Clostridioides difficile* infection. *Clin Infect Dis.* 2024:ciae437. doi: https://doi.org/10.1093/cid/ciae437. PMC11797394 39180326

[23] Tariq R , Pardi DS, Khanna S. Resolution rates in clinical trials for microbiota restoration for recurrent *Clostridioides difficile* infection: an updated systematic review and meta-analysis. Therap Adv Gastroenterol. 2023;16:17562848231174293. doi: https://doi.org/10.1177/17562848231174293 PMC10236242 37274301

[24] DerSimonian R , Laird N. Meta-analysis in clinical trials. Control Clin Trials. 1986;7(3):177-188. doi: https://doi.org/10.1016/0197-2456(86)90046-2 3802833

[25] Tariq R , Syed T, Yadav D, et al Outcomes of fecal microbiota transplantation for *C. difficile* infection in inflammatory bowel disease: a systematic review and meta-analysis. J Clin Gastroenterol. 2023;57(3):285-293. doi: https://doi.org/10.1097/MCG.0000000000001633 34864789

[26] Khoruts A , Rank KM, Newman KM, et al Inflammatory bowel disease affects the outcome of fecal microbiota transplantation for recurrent *Clostridium difficile* infection. Clin Gastroenterol Hepatol. 2016;14(10):1433-1438. doi: https://doi.org/10.1016/j.cgh.2016.02.018 26905904 PMC5552196

[27] Chen T , Zhou Q, Zhang D, et al Effect of faecal microbiota transplantation for treatment of *Clostridium difficile* infection in patients with inflammatory bowel disease: a systematic review and meta-analysis of cohort studies. J Crohns Colitis. 2018;12(6):710-717. doi: https://doi.org/10.1093/ecco-jcc/jjy031 29528385

[28] Lee C , Louie T, Bancke L, et al Safety of fecal microbiota, live-jslm (REBYOTA^™^) in individuals with recurrent *Clostridioides difficile* infection: data from five prospective clinical trials. Therap Adv Gastroenterol. 2023;16:17562848231174277. doi: https://doi.org/10.1177/17562848231174277 PMC10272687 37333464

[29] Qazi T , Amaratunga T, Barnes EL, Fischer M, Kassam Z, Allegretti JR. The risk of inflammatory bowel disease flares after fecal microbiota transplantation: systematic review and meta-analysis. Gut Microbes. 2017;8(6):574-588. doi: https://doi.org/10.1080/19490976.2017.1353848 28723262 PMC5730391

[30] Allegretti JR , Mehta SR, Kassam Z, et al Risk factors that predict the failure of multiple fecal microbiota transplantations for *Clostridioides difficile* infection. Dig Dis Sci. 2021;66(1):213-217. doi: https://doi.org/10.1007/s10620-020-06198-2 32170474

[31] Kelly CR , Fischer M, Allegretti JR, et al ACG clinical guidelines: prevention, diagnosis, and treatment of *Clostridioides difficile* infections. Am J Gastroenterol. 2021;116(6):1124-1147. doi: https://doi.org/10.14309/ajg.0000000000001278 34003176

[32] Peery AF , Kelly CR, Kao D, et al; AGA Clinical Guidelines Committee. AGA clinical practice guideline on fecal microbiota-based therapies for select gastrointestinal diseases. Gastroenterology. 2024;166(3):409-434. doi: https://doi.org/10.1053/j.gastro.2024.01.008 38395525

