# Supplementary material for: Safety and Efficacy of Fecal Microbiota, Live-jslm (REBYOTA®), for the Prevention of Recurrent Clostridioides difficile Infection in Participants With Inflammatory Bowel Disease in PUNCH CD3-OLS
Source: Inflamm Bowel Dis. 2025 Jan 25;31(8):2112–22. doi: 10.1093/ibd/izae291 (PMC12342783; doi:10.1093/ibd/izae291)
Supplement: izae291_suppl_Supplementary_Tables_S1-S2 [file izae291_suppl_supplementary_tables_s1-s2.docx]

**SUPPLEMENTARY MATERIAL**

**Supplementary Table 1.** Summary of TEAEs within 8 weeks and between 8 weeks and
6 months of second course of RBL (safety population).

|  | **Within 8 Weeks** | | **8 Weeks to 6 Months** | |
| --- | --- | --- | --- | --- |
|  | **IBD^a^ N = 9** | **No IBD N = 112** | **IBD^a^ N = 9** | **No IBD N = 112** |
|  | **Participants (% of participants)**  **[events]** | | | |
| **Any TEAE** | 2 (22.2)  [2] | 45 (40.2)  [103] | 1 (11.1)  [1] | 18 (16.1)  [64] |
| **TEAEs by maximum severity** | | | | |
| Mild | 2 (22.2)  [2] | 19 (17.0)  [52] | 1 (11.1)  [1] | 4 (3.6)  [25] |
| Moderate | 0 | 21 (18.8)  [45] | 0 | 12 (10.7)  [37] |
| Severe | 0 | 5 (4.5)  [6] | 0 | 2 (1.8)  [2] |
| Potentially life-threatening | 0 | 0 | 0 | 0 |
| **Any serious TEAE** | 0 | 3 (2.7)  [4] | 0 | 3 (2.7)  [3] |
| **Serious TEAEs by relatedness^b^** | | | | |
| Related to RBL | 0 | 0 | 0 | 0 |
| Related to administration procedure | 0 | 0 | 0 | 0 |
| Related to CDI | 0 | 2 (1.8)  [3] | 0 | 0 |
| Related to a preexisting condition | 0 | 1 (0.9)  [1] | 0 | 1 (0.9)  [1] |
| **TEAEs leading to withdrawal from study** | 0 | 0 | 0 | 0 |
| **TEAEs leading to death** | 0 | 0 | 0 | 0 |

^a^All TEAEs in the IBD group following a second course of RBL were reported by participants with UC.

^b^Relatedness categories are not mutually exclusive.

Abbreviations: CDI, *Clostridioides difficile* infection; IBD, inflammatory bowel disease; RBL, fecal microbiota, live‑jslm; TEAE, treatment-emergent adverse event; UC, ulcerative colitis.

**Supplementary Table 2.** Summary of treatment outcomes following second course of RBL (mITT population).

|  | **IBD**  **N = 9** | **CD**  **N = 3** | **UC**  **N = 6** | **No IBD**  **N = 112** |
| --- | --- | --- | --- | --- |
|  | **Participants, n (%)** | | | |
| **Treatment success at 8 weeks** | | | | |
| Treatment success^a^ | 2 (22.2) | 1 (33.3) | 1 (16.7) | 65 (58.0) |
| Treatment failure | 3 (33.3) | 1 (33.3) | 2 (33.3) | 26 (23.2) |
| Indeterminate^b^ | 4 (44.4) | 1 (33.3) | 3 (50.0) | 21 (18.8) |
| **Sustained clinical response at 6 months^c^** | | | | |
| Yes | 2 (100) | 1 (100) | 1 (100) | 57 (87.7) |
| No | 0 | 0 | 0 | 7 (10.8) |
| Indeterminate^b^ | 0 | 0 | 0 | 1 (1.5) |

^a^Treatment success is defined as the absence of CDI-related diarrhea through 8 weeks after the second course of RBL.

^b^Efficacy outcome was indeterminate if CDI test was inconclusive or missed at time of visit.

^c^The percentage of participants with sustained clinical response at 6 months is calculated with the number of participants with treatment success as the denominator.

Abbreviations: CDI, *Clostridioides difficile* infection; CD, Crohn’s Disease; IBD, inflammatory bowel disease; RBL, fecal microbiota, live‑jslm; mITT, modified intent-to-treat; UC, ulcerative colitis.
